# The online and offline effects of changing movement timing variability during training on a finger-opposition task

**DOI:** 10.1038/s41598-022-16335-8

**Published:** 2022-08-03

**Authors:** Jason Friedman, Assaf Amiaz, Maria Korman

**Affiliations:** 1 Department of Physical Therapy, School of Health Professions, Sackler Faculty of Medicine, Tel Aviv University, Tel Aviv, Israel; 2 Sagol School of Neuroscience, Tel Aviv University, Tel Aviv, Israel; 3 Department of Occupational Therapy, Faculty of Health Sciences, Ariel University, Ariel, Israel

**Keywords:** Human behaviour, Motor control

## Abstract

In motor learning tasks, there is mixed evidence for whether increased task-relevant variability in early learning stages leads to improved outcomes. One problem is that there may be a connection between skill level and motor variability, such that participants who initially have more variability may also perform worse on the task, so will have more room to improve. To avoid this confound, we experimentally manipulated the amount of movement timing variability (MTV) during training to test whether it improves performance. Based on previous studies showing that most of the improvement in finger-opposition tasks comes from optimizing the relative onset time of the finger movements, we used auditory cues (beeps) to guide the onset times of sequential movements during a training session, and then assessed motor performance after the intervention. Participants were assigned to three groups that either: (a) followed a prescribed random rhythm for their finger touches (Variable MTV), (b) followed a fixed rhythm (Fixed control MTV), or (c) produced the entire sequence following a single beep (Unsupervised control MTV). While the intervention was successful in increasing MTV during training for the Variable group, it did not lead to improved outcomes post-training compared to either control group, and the use of fixed timing led to significantly worse performance compared to the Unsupervised control group. These results suggest that manipulating MTV through auditory cues does not produce greater learning than unconstrained training in motor sequence tasks.

## Introduction

Skilled motor performance is obtained through repetitive practice that drives improvements in smoothness, speed and accuracy of movement execution^1–3^. However, in terms of their kinematic^4^ and kinetic^5^ characteristics, repetitions of the same movements are never identical; this phenomenon is called motor or movement-to-movement variability. Several different processes have been suggested to explain the omnipresence of motor variability in simple and complex movements^6^. First, the motor commands originate from inherently noisy sensory-motor processes integrating central and peripheral neural signals into muscle force^7–10^. Motor variability is a consequence of this noise and, thus, an error marker of the system, i.e., the mechanisms responsible for muscle activations are inherently inaccurate. According to this view, motor variability is an often undesirable characteristic of motor performance^7,10,11^. Nevertheless, a complementary and not mutually exclusive notion regards the functional role of motor variability, which suggests that it is generated by the central nervous system to foster the exploration of the large number of possible solutions of motor control in a given task^3,12^. One widely accepted notion is that variability decreases with motor learning^13^.

Motor redundancy—many degrees of freedom in the human body (joints and muscles)—is a key adaptive characteristic of the motor system enabling multiple movement solutions for a given task^14,15^. Motor variability subserves this flexibility in reaching an optimal solution among many possible alternatives in a cost-effective way^16–18^, a notion that is conceptualized as structure learning of a motor task that limits movements to a subspace of all possible movements^19^. Accordingly, amplification of variability may promote motor learning through action exploration^12,20^. It was proposed that motor variability is actively modified through learning leading to decreased task-relevant variability while task-irrelevant variability remains high, resulting in gains in performance accuracy^16,21^.

The experimental evidence on the role of variability in predicting inter-individual differences in motor learning is rather inconclusive. Since the publication of “schema theory” by Schmidt^22^ that suggested that variable practice benefits motor schema development compared to constant practice, the supportive evidence was inconsistent either with adults or with children (see meta-analysis by Van Rossum^23^). The benefits of task-relevant trial-to-trial motor variability (as opposed to practice variability) during skill acquisition were shown in both animal, e.g., songbird models^24^, and humans^3^, providing evidence that motor variability increases the speed of motor learning. In particular, Wu and colleagues found that the magnitude of task-specific initial motor variability correlated with the rate of improvement for both reward-based reinforcement learning and error-based motor adaptation^3^, although other studies have not found this effect, for example in a study looking at motor variability before learning, they found that initial task-space variability did not predict ability to learn new movements^25^. More recent studies suggest that motor variability can have both negative and positive effects on learning. Using computational modelling, He and colleagues showed that variability can have positive, neutral, or negative effects on adaptation learning, and demonstrated this in a series of experiments and a meta-analysis of previous studies^26^. Singh and colleagues found that in a redundant reaching task, task-space variability had low correlation with learning, whereas null-space variability (defined as variability which does not affect the task) did correlate with learning^27^. It is clear from these studies that variability is certainly not just a noise detrimental to motor performance that needs to be suppressed. In a recent study, Cardis et al.^28^ used a redundant bimanual shuffleboard task (the goal is to slide a virtual puck as close as possible to a target) to explore the effects of introducing either null or task space variability. They found that variability led to increased exploration of new solutions, but negatively impacted the retention of the learned solution. Higher externally introduced variability was shown to hamper motor learning, regardless of whether it affects task performance; the amount of variability was a more important factor than the dimension in which variability was manipulated^28^. The majority of studies using initial motor variability to examine inter-individual differences in learning have primarily utilized adaptation tasks^3,26,27^; adaptation learning in ecological settings is less common compared to skill learning^13,29^, for example, sequential movement tasks. The role of motor variability in motor sequence learning has been convincingly demonstrated in songbirds^6,24,30,31^ but is an under-researched subject in humans.

Methodologically, variability is mainly studied as an innate characteristic of movements. Nevertheless, it is possible that other factors explain the course of motor learning and that these factors correlate with movement variability. For example, participants with initially low performance level are likely to have high movement variability. They may show better learning relative to their baseline than others just due to the fact that they started lower and therefore have more room for improvement, and not because of higher movement variability^32,33^. Systematically controlling the variability during training by enhancement or suppression in an experimental setup may help us resolve whether increased variability indeed leads to better motor learning.

It is of theoretical and practical importance to understand how variability during training affects the multi-stage process of post-training motor memory consolidation. The time-course of skill acquisition, or more accurately, evolution of skill-learning task performance, can be characterized by at least two distinct phases: a fast, online, phase of performance improvement, and a delayed, offline, time-dependent improvement phase occurring between training sessions^34–37^. The latter phase has been conceptualized as reflecting memory consolidation processes^38^. The conjecture is that in perceptual and motor tasks the process of memory consolidation can be triggered by the training experience under certain conditions and requires time to become effective in terms of performance. Multiple factors determine whether successful consolidation of a training experience will occur, e.g., training structure, feedback, sleep pre- and post-training^2,39–42^. Among them, self-regulation during training, which inherently includes self-generated movement timing variability (MTV), i.e., the natural variance that occurs when the participants are not explicitly told which timing to use to perform the sequence “as fast and as accurate” as they can, was also recognized as a beneficial factor in the context of sequence learning^2^, although it is not known whether this is the optimal type of MTV. In motor sequence learning, different kinematic characteristics of the finger movements may independently change following training and exhibit a specific time-course of learning-related modifications, as well as generalization^2^. Long-term offline, consolidation gains in performance speed were shown to be mainly due to reducing gaps between finger movements in different types of self-regulation allowed during training (e.g., paced for every movement in the sequence, cued only for the initiation of the sequence or self-initiated sequence execution, i.e., continuous repeated sequence execution in the 30 s time window), with self-initiated training inducing higher learning gains. Based on these observations, we aimed to introduce quantifiable and harmonized across participants variability in terms of the time between finger touches, as this may lead to better selection of the timing between movements, which is where we expect most of the improvement to come from.

The focus in the current study is on the effects of temporal variability of a sequence of movements during training on immediate and delayed motor learning outcomes. In particular, we examined the influence of experimentally controlled Movement Timing Variability (MTV) of within-sequence movement structure in comparison to self-generated MTV on the time-course of finger-to-thumb opposition sequence (FOS) task learning over a 24 h interval. The primary outcome measures included: (1) during training session, the “reaction time”—the time between the last beep of the instructed rhythm and the finger movement, (2) the MTV of the differences between finger-to-thumb touch times, (3) improvement in number of correct sequences performed in 30 s, both within-session (online, on the same day), and between-session (offline, tested 24 h later). We directly compared three training protocols that differed only in within-training acoustically delivered sequence execution instructions. One training condition was based on imposed pseudo-random MTV (Variable group). The other two groups served as control groups: one group received fixed-rate MTV (Fixed control group), while the other control group (Unsupervised control group) was not given instructions about timing of the individual fingers, only when to start the sequence, i.e., had unsupervised MTV (Unsupervised control group). This group was included as a control as it is similar to the training protocol often used in finger opposition studies (e.g.^37,43^). During the training session, the same five-element sequence of fingers touching the thumb was performed 160 times by participants in all groups. The Fixed control and Variable groups heard before each sequence repetition a rhythmic auditory cue consisting of 5 beeps (constant and variable pace, respectively) instructing the pace in which the sequence of the finger movements should be executed. Participants in the Unsupervised control group, received a single auditory cue after which the entire sequence was performed, a standard and well-researched training paradigm^2,43,44^. During training all groups were afforded 160 repetitions of the trained FOS. No task-relevant feedback (visual or knowledge of results) was available to participants during training and tests, to restrict error-based learning^26^. We hypothesized that high MTV, both imposed (Variable group) and natural (Unsupervised control group), would lead to larger learning gains (i.e., relatively more sequences performed) compared to low MTV (Fixed control group), immediately after training and also offline at 24 h retest. Limited processing capacity, i.e., load on working memory (active storage and manipulation of information^45^), may constrain motor learning and performance^46,47^. Thus, we predicted that the inclusion of supervised MTV in training in the Variable group may benefit some learning processes but concurrently may have costs due to increasing the cognitive load. Specifically, we expected that reaction times to the auditory cues during imposed variable training will be slower compared to fixed imposed or self-controlled rate of movement execution during training.

To examine the effect of MTV in training related differences, we examined how the MTV changes throughout the phases of learning across the groups. We decomposed the variability observed into its underlying kinematic components, and then tested the effect of these differences in variability on the standard outcome measures—improvement in the number of correct sequences performed and the number of errors.

## Results

### Reaction time differences between the groups

**Figure 1:**
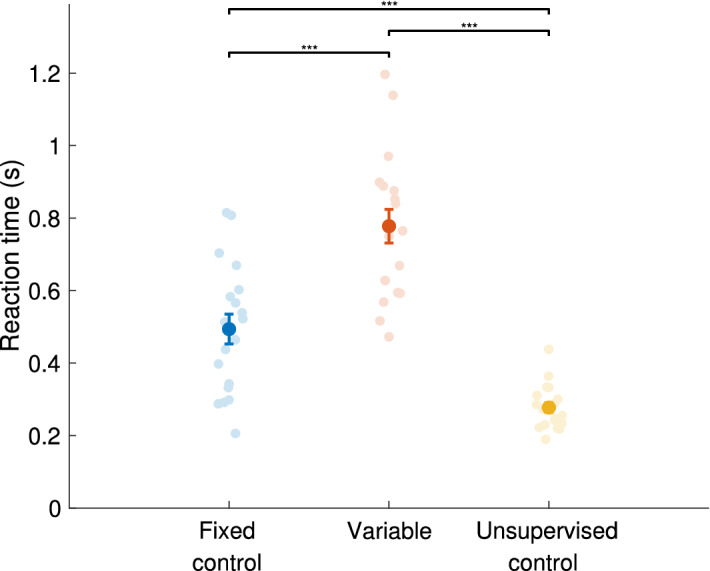
Mean reaction time during the training segment, defined as the time from the last beep to the onset of the first movement. The faint circles are data for all subjects, dark dots are the mean, error bars are the standard error for each group, the black bars indicate significant between-group differences. ***p ≤ 0.001in post-hoc t-tests. N = 19 for all groups.

We first explored the differences in the training session induced by the three different protocols. We observed a difference in the “reaction time” (RT), defined as the time between the last beep and the first movement made, during the training blocks, see Fig. 1. An ANOVA found a main effect of group (F(2,54) = 18.960, p < 0.001), with post-hoc t-tests showing that the RT was the fastest in the Unsupervised control group (0.28 ± 0.01 s), which was significantly faster than the Fixed control group (0.49 ± 0.04 s, t(36) = 5.01, p < 0.001), which was significantly faster than the Variable group (0.78 ± 0.05 s, t(35) = 4.52, p < 0.001).

### Movement Timing Variability (MTV) induced by the training

**Figure 2:**
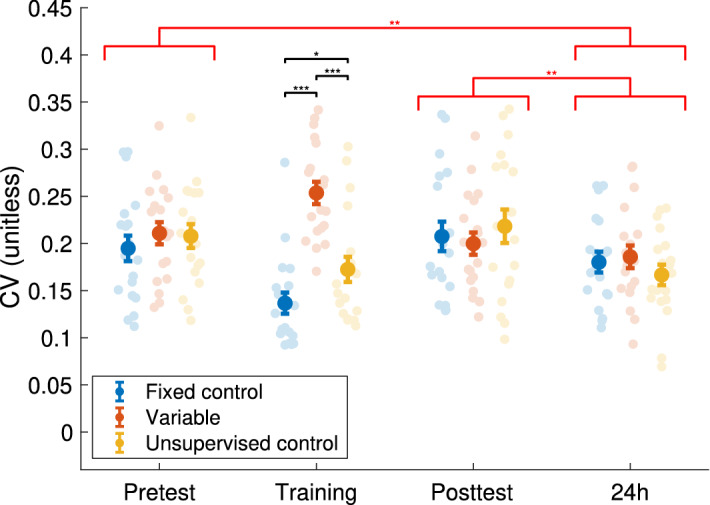
Coefficient of variation (CV) for movement timing variability between touching the thumb and the other finger, for the three groups, averaged across movements in the different blocks. The faint circles are data for all subjects, dark dots are the mean, error bars are the standard error for each group. The black bars indicate significant between-group differences, the red bars indicate significant differences between sessions. *p ≤ 0.05, **p ≤ 0.01, ***p ≤ 0.001. N = 19 for all groups.

Next, we examined how the variability of the differences between touch times (i.e., when the thumb and other finger touched) changed during the different sessions, quantified using the coefficient of variation (CV, the standard deviation divided by the mean), shown in Fig. 2. The CV was used to avoid effects caused by the correlation of mean and standard deviation. A mixed-design ANOVA showed a main effect of session (F(3,162) = 6.906, p < 0.001)—the CV was lower at 24 h (0.178 ± 0.006) compared to both the Pretest (0.203 ± 0.007, t(112) = 2.64, p = 0.01) and the immediate Posttest (0.208 ± 0.009, t(112) = 2.85, p = 0.005), indicating overnight consolidation of the CV. In addition, an interaction of session and group (F(6,162) = 5.610, p < 0.001) was observed. Post-hoc tests showed that during training, the Variable group showed higher CV (0.254 ± 0.012) than the Unsupervised control group (0.172 ± 0.013, t(36) = 4.51, p < 0.001), which showed higher CV than the Fixed control group (0.137 ± 0.011, t(36) = 2.05, p = 0.048). However, this effect was transient—no significant difference was observed between groups in any other session (t-tests showed all p > 0.05). Importantly, at baseline (Pretest), there were no significant differences in CV between the groups.

There are three possible ways that an increase in CV may be achieved during training—(a) by increasing variability in the time taken to move the finger, (b) by increasing variability in the time the fingers touch, and (c) by increasing variability in the pauses between the movements. These three potential explanations are explored in the next section.

### Variance of the decomposed sequence

**Figure 3:**
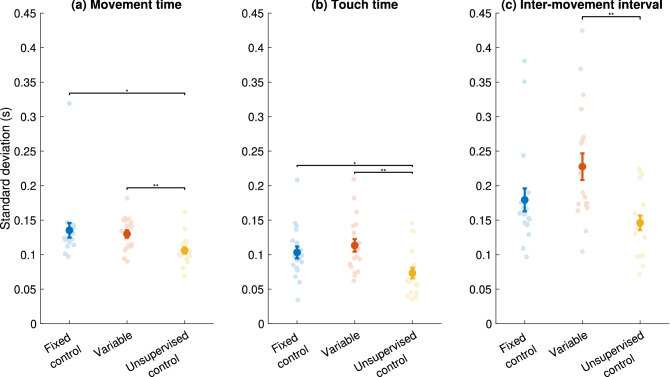
Differences in standard deviation across groups during training. Each panel shows a different component of the movement. The faint circles are data for all subjects, darker dots are the mean, error bars are the standard error for each group. The black bars indicate significant between-group differences. *p ≤ 0.05, **p ≤ 0.01. N = 19 for all groups.

To further examine the differences in movement timing variability induced in the three groups, we decomposed the time taken to perform each sequence in the training session and calculated the standard deviation of each of the three parts, shown in Fig. 3. It is important to examine the components individually because as we previously showed^2,44^, the time course of learning, as well as the overall contribution to improvement in performance, is not the same across different components. Specifically, the total time for a sequence was decomposed into the movement time (the time taken for the appropriate finger to move towards the thumb), the touch time (how long the finger and thumb touch), and the inter-movement interval (the time between releasing the touch, and when the next finger starts moving). Full definitions are given in the methods section. For the decomposition into parts, we used standard deviation rather than CV because the values for the inter-movement interval approach zero for some participants, which leads to very large values for the CV. To examine the effects of training type, we performed a one-way ANOVA on the three components during training.

For all three components, a main effect was observed for the group. In terms of movement time (F(2,54) = 4.413, p = 0.017), post-hoc tests showed that the standard deviation was greater than the Unsupervised control group (0.106 ± 0.005 s) for both the Fixed control (0.135 ± 0.011 s; p = 0.03) and Variable (0.130 ± 0.005 s, p = 0.006) groups. Similarly, for the touch time (F(2,54) = 4.413, p = 0.017), post-hoc tests showed that the standard deviation was greater than the Unsupervised control group (0.073 ± 0.008 s) for both the Fixed control (0.103 ± 0.009 s; p = 0.029) and Variable (0.113 ± 0.009 s, p = 0.006) groups. For the inter-movement interval (F(2,54) = 6.570, p = 0.003), only the Variable group (0.228 ± 0.019 s) showed a standard deviation significantly greater than the Unsupervised control group (0.146 ± 0.011 s, p = 0.002).

Parallel analyses were performed for the main outcome measures of performance speed and accuracy themselves (and not their variability), as described below.

### Improvement in number of correct sequences performed

**Figure 4:**
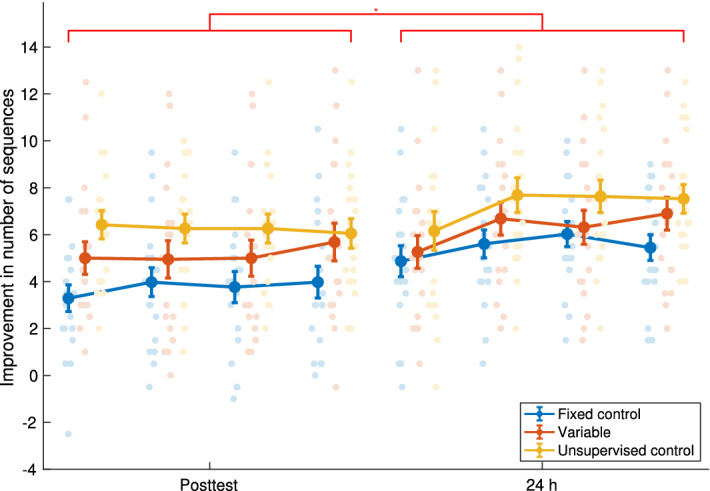
Improvement in number of sequences (relative to the mean number performed in the first two blocks of the Pretest). The faint dots are the values for all subjects (for each of the four Pretest or Posttest trials), the darker lines and error bars are the mean ± standard error. The red bar indicates a significant between-session difference. A main effect of group was also observed: the Unsupervised control group showed significantly greater improvement than the Fixed control group. N = 19 for all groups.

The relative online (Posttest) and offline, overnight (24 h test) improvements in performance speed (increase in the mean number of correct sequences performed in 30 s test blocks relative to the mean number performed in the first two blocks of the Pretest) is shown in Fig. 4. A main effect of session was observed (F(1,53) = 5.886, p = 0.019), with higher performance at 24 h (6.34 ± 0.36 sequences) than at Posttest (5.05 ± 0.37 sequences). A main effect of group was also observed (F(2,53) = 3.694, p = 0.031), but no interaction. There was also no main effect of reaction time (F(1,53) = 0.473, p = 0.494). Post-hoc tests showed that the Unsupervised control group showed significantly more improvement (6.75 ± 0.56 sequences) than the Fixed control group (4.62 ± 0.49 sequences, p = 0.032), but the differences between the Fixed control and the Variable (5.72 ± 0.65 sequences, p = 0.353) and between the Unsupervised control and the Variable groups were not significant (p = 0.353). These results were supported by also performing a Bayesian repeated measures ANOVA, see Table 1. The best model for describing the data was the same as found in the non-Bayesian ANOVA (i.e., a main effect of session and group, but no interaction or main effect of RT).

**Table 1:** Model comparison for improvement in number of correct sequences performed (Bayesian repeated measures ANOVA).

Model comparison	P (M)	P (M|data)	BF_M_	BF_10_	Error %
Models					
Null model (incl. subject)	0.100	4.844 × 10^− 5^	4.359 × 10^− 4^	1.000	
Session + group	0.100	0.389	5.719	8021.513	2.404
Session + group + RT	0.100	0.200	2.252	4131.570	2.733
Session	0.100	0.191	2.120	3936.827	2.344
Session + group + session*group	0.100	0.089	0.884	1847.144	2.814
Session + RT	0.100	0.081	0.793	1672.145	2.581
Session + group + RT + session*group	0.100	0.050	0.474	1032.395	3.841
Group	0.100	9.716 × 10^− 5^	8.745 × 10^− 4^	2.006	1.300
Group + RT	0.100	4.483 × 10^− 5^	4.035 × 10^− 4^	0.926	2.273
RT	0.100	1.848 × 10^− 5^	1.663 × 10^− 4^	0.382	2.079

Post-hoc Bayesian t-tests (see Table 2) similarly showed strong evidence for differences between Fixed control and Unsupervised control groups (BF_10_ = 58), but only anecdotal evidence for differences between the other combinations.

**Table 2:** Bayesian t-test post-hoc comparisons for improvement in number of correct sequences performed (by group).

	Prior odds	Posterior odds	BF_10,U_	Error %
**Fixed control**				
Variable	0.587	0.528	0.898	0.012
**Fixed control**				
Unsupervised control	0.587	34.286	58.368	4.297 × 10^− 8^
**Variable**				
Unsupervised control	0.587	0.401	0.683	0.013

The results in terms of number of correct sequences (rather than relative improvement) are presented in the Supplementary Material (SM-1).

We note that nearly all subjects improved as a result of the training, and at Posttest the improvement was significantly greater than 0 (i.e., all groups improved during training), as shown by a one-sample t-test (t(56) = 13.619, p < 0.001).

We compared the errors at each of the three time points using the Kruskal Wallis test (as many subjects made no errors, the distributions cannot be normal). We found a significant difference only at Posttest (H(2) = 7.209, p = 0.027). Post-hoc Mann–Whitney U-tests found that the Unsupervised control group (median 0.00, IQR 0.44) showed significantly less errors than the Variable group (median 0.75, IQR 0.75; U = 459, p = 0.0083).

### Decomposition of time taken to complete sequence

**Figure 5:**
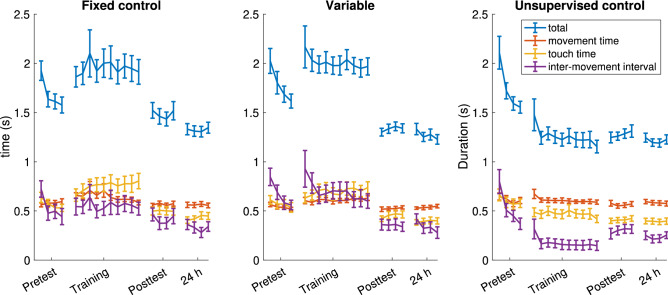
Decomposition of the sequence duration into parts. Each point is the mean value of the quantity in a given trial (4 trials during Pretest, Posttest and 24 h, 10 trials during training). Note that the total time does not include inter-movement interval between sequences, because the sequences were not performed continuously during training. Values shown are the means, error bars are the standard error.

We decomposed the movement time into four parts, see Fig. 5, into movement time (the time the finger closes towards the thumb), touch time (when the finger is touching the thumb), and the inter-movement time (the times between finishing moving the previous finger, and starting to move the next finger). The inter-movement time was divided into the time between movements in the same sequence, and between sequences.

A mixed-design ANOVA was performed on the four quantities (including between-movement intervals, not shown in Fig. 5) on the three test sessions, not including the training. A main effect was observed for session for all four quantities (movement time: F(2,108) = 3.067, p = 0.05; touch time: F(2,108) = 65.506, p < 0.001; inter-movement interval within sequence: F(2,108) = 71.821, p < 0.001; inter-movement interval between sequences: F(2,106) = 34.458, p < 0.001).

For the movement times the participants were faster during the Posttest (0.553 ± 0.086 s) compared to the Pretest (0.578 ± 0.099 s; p = 0.049). The touch times were similarly faster during the Posttest (0.091 ± 0.029 s) compared to the Pretest (0.116 ± 0.036 s, p < 0.001), in addition, there was a further reduction at 24 h (0.081 ± 0.026 s, p = 0.003). The inter-movement interval within the sequence were faster at Posttest (0.357 ± 0.228 s; p < 0.001) and 24 h (0.301 ± 0.204 s; p < 0.001) compared to Pretest (0.582 ± 0.292 s). Similarly, the inter-movement intervals between sequences were also faster at Posttest (0.154 ± 0.063, p < 0.001) and 24 h (0.135 ± 0.050, p < 0.001) compared to Posttest (0.254 ± 0.149 s).

No main effect of group was observed for any of the four measures, while an interaction of session and group was observed only for the inter-movement interval within sequence (F(4,108) = 2.437, p = 0.05). Post-hoc tests showed that while all groups showed a significant improvement (a decrease) in inter-movement interval from Pretest to Posttest (Fixed control: 0.129 ± 0.200 s, p = 0.011; Variable: 0.315 ± 0.220 s, p < 0.001; Unsupervised control: 0.230 ± 0.193 s, p < 0.001) and during consolidation a significant improvement (i.e., greater than zero) was shown for the Fixed control (0.086 ± 0.113 s, p = 0.004) and Unsupervised control groups (0.066 ± 0.105 s, p = 0.013), but not for the Variable group (0.015 ± 0.120 s, p = 0.609), although the effect sizes were small.

## Discussion

Acquisition of a new motor skill often is measured as a more fluent and faster execution of a task without sacrificing accuracy. The role of movement timing variability (MTV) in within-training and post-training motor sequence learning is largely unknown. The current study provides several novel empirical observations on the matter through comparison between training outcomes under conditions of supervised and unsupervised MTV in different groups of young adult participants practicing a novel 5-element finger-opposition sequence of movements. Two supervised MTV training conditions relied on the affordance of an instructional 5-element auditory rhythmic sequence, fixed or variable, heard before each movement sequence repetition. The Unsupervised control group received a single auditory cue indicating the start of the whole sequence without specification of the rhythm of finger movements.

Our findings suggest that supervised MTV induced high cognitive load, evident in significantly slower RTs (time from the last beep to the onset of the first sequence movement) in the Fixed control and Variable groups compared to the Unsupervised control group. Moreover, the supervised groups also differed from each other: the Fixed control group had significantly faster RTs than the Variable group, likely due to the difference in predictability of the sequence’s pace (Fig. 1). Training in supervised conditions in the current protocol is essentially imposing dual-tasking (a classical paradigm to assess the impact of cognitive load on performance) by demanding to keep the mental representation of the auditory sequence in working memory and to apply it to the execution of finger movements. Slower RTs in supervised MTV conditions are consistent with prior findings, e.g., dual-tasking during sequence execution leading to increased sequence element duration^48^. A recent study, however, found that the learning gains in performance were not deteriorated by the dual-tasking^49^. In the light of this finding, we assume that the effects of supervised MTV and the effects of dual-tasking in the current study could be independent. However, this assumption should be empirically tested in future studies.

Auditory instruction for the three MTV training conditions differentially impacted the variability during the training session, as expected (Fig. 2): the coefficient of variation (CV) of the timing of finger touching events during the training session was higher in the Variable group than in the Unsupervised control group, which showed higher CV than the Fixed control group. However, this effect was transient—no significant differences in the CV were observed between groups even immediately post-training. Over the consolidation period, all three groups showed a significant reduction in CV, however, without between-group differences. Previous studies have also shown that variability reduces over time^21^. These results suggest that differences in MTV during training had no direct impact on the MTV of consolidated skill performance.

The imposed instructions in the Variable group robustly caused an increase in variability in a task-relevant parameter (inter-movement intervals, or gaps within-sequences), which has been previously shown to be responsible for most of the observed improvements in performance with training^2,44^. Despite this increase in task-relevant variability, participants did not improve more than self-paced participants post-training. Rather, at the level of sequence performance, the Unsupervised control group showed the highest gains in speed at 24 h post-training, the differences between the Fixed control and the Variable and between the Unsupervised control and the Variable groups were not significant. When we consider the Fixed condition as a control to the Variable group, we did not observe a significant difference in improvement following the training. In contrast, there was a significant difference between the Unsupervised control and Fixed groups, suggesting that these two groups define two extremes of magnitude of MTV in this protocol, as intended.

Decomposition of the movements into four kinematic components (finger movement time, touch time, and the inter-movement time between movements in the same sequence, and between sequences)^44^, showed that all the components contributed to the improvement, with the largest improvement in inter-movement interval within a sequence (see the Supplementary Material SM-2). This component also improved further in overnight consolidation, except for the Variable group. After 24 h, all groups showed a reduction in variability, as has been observed previously in learning tasks.

As reaction time may be a proxy for cognitive load, we included reaction time as a covariate in analysis of the improvement and number of sequences performed, but we did not find a significant effect of reaction time on the outcomes. Thus while the reaction times differed across groups, within groups reaction time did not predict performance, although to test this more thoroughly a larger sample size would be preferable.

Previous studies have performed manipulations that may not have been described as variability manipulations but were in fact (e.g., self-paced vs. evenly-paced), and showed that the long-term learning outcomes may differ^2^ as a function of movement timing control during training. In contrast to this and other, intentionally targeting movement variability studies^3,25^, here we manipulated the variability through instruction rather than comparing the effect of baseline variability on performance. In this way, we attempted to avoid the confound of subjects that have high variability are likely to have low baselines^6,50^ and thus more room for improvement. An unavoidable limitation of the current protocol is that this intervention changed the nature of the task between groups, which may have partially been responsible for the relative lack of improvement for the Variable group. Additionally, the same relative timing was used for all subjects in the Variable group. This may explain the relative disadvantage of imposing variable MTV—such training could be very different from the pre-existing coordination strategy and also could interfere with the process of gradual evolution of the innate, pre-existing coordination strategy into new motor patterns, as normally happens in the Unsupervised control condition (see Fig. 5). An additional limitation of the study is that we did not test the baseline cognitive or motor differences between the participants.

There is mounting evidence that the role of motor variability depends on the way the variability is induced (e.g., in the task space—affecting task performance or in the null space -not affecting performance, naturally occurring or imposed^27,51–53^). Current findings on the imposed temporal variability are in line with the notion that variability is a complex construct^26,53^ and that changing the task demands via extrinsically induced movement timing variability should be carefully considered in terms of both the immediate and delayed effects on motor learning and memory.

The broader practical implications of the current study are that development of motor training programs for typical and special populations should take into account the temporal structure of the instructions given, as the temporal organization type of the instructions can impact the learning outcomes even when otherwise the same amount of practice is performed. This was demonstrated in this and other studies^2,54–56^.

Altogether, our findings provide empirical evidence for the important role of unconstrained and unsupervised temporal structure of action exploration during novel motor skill acquisition. Higher MTV during training may impact performance improvement across the consolidation phase, but imposed temporal variance in the FOS task does not necessarily improve performance. Rather, it seems that allowing self exploration of the sequence is preferential for improved learning in this task^57^. It may be that by using natural variability, there will be a gradual change in how the sequences are performed, which starkly contrasts with the abrupt changes in variability used in this study, which did not seem to help improve motor learning. Evenly paced temporal instructions during sequence training seem to be undesirable.

## Methods

### Participants

Fifty-eight right-handed participants from the Tel Aviv University student population took part in the experiment. Right-handedness was confirmed using the Edinburgh Handedness Inventory^58^. Ethics approval was received from the Tel Aviv University Institutional Review Board, and all experiments were performed in accordance with the relevant guidelines and regulations. The participants signed an informed consent form before beginning the experiments, and were paid 70 shekels (approximately $20) for their participation.

As a result of failures in the sensor measurements (sensors falling off the hand), one participant was removed from the Fixed control group, Therefore, only 57 subjects (18 males and 39 females, average age 25.6 ± 2.5 years, range 22–33 years) were included in the final analyses. The number of participants recruited was based on previous, similar studies^43,44^ comparing groups in sequence-learning tasks.

### Experiment protocol

**Figure 6:**
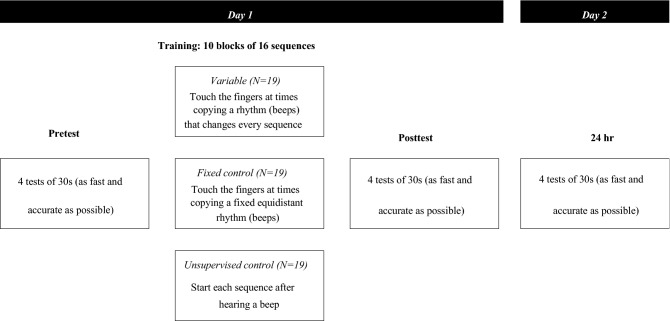
Experimental protocol. The groups differed only during the training.

The protocol is shown in Fig. 6. Participants were randomly assigned to one of three different groups—Variable, Fixed control or Unsupervised control, which differed only during the training session.

**Figure 7:**
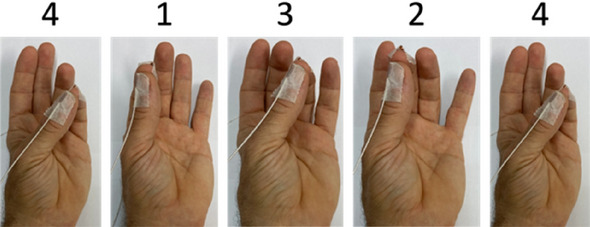
The sequence of finger-to-the thumb opposition movements (4–1–3–2–4) that participants were required to perform (from left to right) throughout the experiment. During tests the participants were instructed to perform the sequence repeatedly as “fast and as accurate” as they can during a 30 s interval. During training the participants of the Fixed control and Variable groups were following an auditory guide (set of rhythmic beeps) for the timing of the within-sequence movements, while the participants of the Unsupervised control group had an auditory cue only for the start of the sequence. The wires shown are the Ascension trakSTAR sensors used to measure the 3D location of the fingertips.

The participants were required to perform a finger-opposition task^43^, in which the thumb and another finger of the left, non-dominant hand were required to touch in a given sequence (4–1–3–2–4): 1 corresponds to the index finger touching the thumb, 2 to the middle finger touching the thumb, etc., see Fig. 7. The primary outcome measure in this task is the improvement in the number of correct sequences than can be performed in a 30 s test (from before to after training). The experimenter demonstrated touching the thumb and finger but did not demonstrate the sequence. The participants were instructed not to look at their fingers while performing the task. After successfully performing the sequence 3 times, the recordings started. In the first session, the participants first performed four test trials (Pretest). Each trial was 30 s long, and the participants were instructed to perform accurately as many sequences as possible during this time. Beeps indicated the start and end of the trial, and a 30 s rest period was provided between trials. Before each trial, the sequence was shown on the screen as text (e.g., 4–1–3–2–4). During the tests the screen was blank.

Following a 5-min break, the participants performed ten training trials, with each trial consisting of sixteen repetitions of the sequence. Participants in the Variable and Fixed control groups heard a rhythmic sequence of 5 beeps. Immediately after hearing the beeps, they needed to produce the sequence with their fingers touching the thumb in the same rhythm as the beeps they just heard (but without hearing the beeps again). As the duration for performing a sequence at baseline varies greatly across participants (see Fig. S1 in Supplementary Materials), the total duration of the sequence was selected to be identical to the mean duration of the sequences produced by the participant in the fourth block of the Pretest. This was selected so that the speed would be feasible for the subject, but still somewhat challenging. For the Variable group, the duration between the first four beeps were pseudorandomly selected from a normal distribution with a mean of 25% of the sequence time, and a standard deviation of 5% of the sequence time. The duration between the fourth and fifth beeps was then the remaining time left in the sequence—unless it was below 10% or above 40% of the total sequence time, in which case it was rejected. The same relative timing was used for all subjects in the Variable group. For the Fixed control group, the duration between the beeps was fixed (25% of the sequence time). The participants were instructed to copy the rhythm and focus on accuracy. For the Unsupervised control group, a single beep was played, after which they needed to produce the entire sequence. For all groups, the same type of beep was used (500 Hz beep for 50 ms).

Following the training (after a 2 min break), each participant performed four test trials (Posttest), with the same instructions as for the Pretest. The session on the first day took approximately 30 min in total. Approximately 24 h after the initial training session, participants were re-tested on performing the same trained sequence (24 h). The session on the second day took approximately 10 min.

### Measurement and data pre-processing

The finger movements of the participants were recorded using an Ascension trakSTAR magnetic motion capture system, sampling at 240 Hz. The six sensors were taped on each fingertip and one on the palm of the left hand. The sensors were taped on the fingertips such that no tape was on the finger pads, in order to preserve full touch sensation at the fingertips. The experiments were run using the “Repeated Measures” software^59^, Matlab (Mathworks, Inc.) software that runs on top of the Psychtoolbox^60^.

Data were analyzed offline using custom Matlab software. The raw data was low-pass filtered with a 4th order two-way Butterworth filter, with a cutoff frequency of 20 Hz. Finger touches (of the thumb and other fingers) were identified automatically based on the minimal distances between the thumb and other fingers. The timing of these touches was manually corrected (using a custom graphical user interface developed in Matlab) so that the number of sequences and errors performed matched the number recorded by the experimenter (from observation during the experiment). Each sequence was then decomposed to determine the relative contribution of the different temporal parts, following the technique used in Friedman and Korman^2,44^, based on the distances between the thumb and the relevant finger. The movement time was defined as the time from the last trough in the derivative of the finger distance (i.e., when the finger and thumb start moving closer together) to the moment they touch. The touch times (when the thumb was touching the other finger) were defined as the time adjacent to the touch where the magnitude of the derivative of the finger-thumb distance is below 5% of the maximum magnitude of the derivative of the finger-thumb distance. The remaining time (the inter-movement intervals) was divided into the times between movements within a sequence, and between sequences (i.e., between the end of one sequence and the start of the next sequence). Normalized data (relative improvement) were calculated relative to baseline performance in the first two trials of the pre-test. We performed this normalization on both the total time to complete a sequence correctly, and on the components described above (movement times, touch times, inter-movement intervals). We subtracted the duration of the appropriate component from the baseline value (for one sequence).

### Statistical analysis

We used a one-way between-subjects ANOVA to compare the cognitive load (as reflected in the reaction times) across the groups. Then, to determine whether the intervention changed the variability during training, we calculated the coefficient of variation between the finger-to-thumb touch times. The coefficient of variation (CV—the standard deviation divided by the mean) was used rather than the standard deviation to avoid effects caused by the often-observed correlation of mean and standard deviation. We compared the three groups and sessions (Pretest, Training, Posttest and 24 h) using a mixed-design ANOVA.

One-way ANOVAs were used to compare the differences between the three groups in standard deviation of the decomposed quantities (movement time, touch time and inter-movement interval within a sequence). For this analysis, standard deviations were used rather than coefficient of variation because the inter-movement interval approaches 0, which makes the coefficient of variation unstable.

The relative improvement (compared to the Pretest) in number of sequences was compared across groups (Fixed control, Variable and Unsupervised control) and sessions (Posttest and 24 h) using a mixed-design ANOVA. Reaction time was considered as a covariate. Additionally, a Bayesian ANOVA was performed on the same data with the same factors. Similar analyses for number of sequences is presented in the Supplementary Material (SM-1). The changes in the number of errors were compared using a Kruskal Wallis test (as the errors cannot be normally distributed as there is a floor effect of zero errors), followed by post-hoc Mann–Whitney tests.

We also compared the decomposed quantities (movement time, touch time, within-sequence inter-movement interval, and between-sequence inter-movement interval) and not their variability, using one way ANOVAs. Holm corrections were used for multiple post-hoc comparisons, and corrected p-values less than 0.05 were considered significant. Normality of the variables was confirmed visually using a Q–Q plot, and Levene’s test was used to confirm equality of variances (homogeneity). Statistical analyses were performed using JASP^61^.

## Supplementary Information

Supplementary Information.

## Data Availability

We included all the data needed for the evaluation of the conclusions in the Results section or in the Supplementary Information file. Additional data related to this article may be requested from the authors.
