# Supplementary material for: The online and offline effects of changing movement timing variability during training on a finger-opposition task
Source: Sci Rep. 2022 Aug 3;12:13319. doi: 10.1038/s41598-022-16335-8 (PMC9349301; doi:10.1038/s41598-022-16335-8)
Supplement: Supplementary file 1 — Supplementary Information. [file 41598_2022_16335_MOESM1_ESM.docx]

**Supplementary material**

*SM-1. Number of correct sequences*

We complement the presentation of the relative improvement with the number of correct sequences (Figure S1), performing a mixed-design ANOVA, with a between-subject factor of Group (Fixed, Variable and Control), and a within-subject factor of Session (Pretest, Posttest and 24h). A main effect of session was observed (F(2,108)=206.926, p<0.001). Post-hoc tests showed that more sequences were performed in Posttest (17.1 ± 0.4) compared to Pretest (13.1 ± 0.4, p<0.001), and there was an overnight consolidation effect with an increase to 18.4 ± 0.4 sequences (p<0.001). There was no main effect of group (F(2,54)=1.219, p=0.303), although there was an interaction of group and session (F(4,108)=2.487, p=0.048). However, none of the post-hoc tests were significant (all p>0.05).

We also performed a Bayesian ANOVA with the same factors. The best model was with a main factor of Time only (with no interaction), see Table S1.

| Table S1. Model comparison for number of correct sequences performed (Bayesian repeated measures ANOVA) | | | | | | | | | | | |
| --- | --- | --- | --- | --- | --- | --- | --- | --- | --- | --- | --- |
| **Models** | | **P(M)** | | **P(M\|data)** | | **BF_M_** | | **BF_10_** | | **error %** | |
| Null model (incl. subject) |  | 0.200 |  | 7.453e-35 |  | 2.981e-34 |  | 1.000 |  |  |  |
| Time |  | 0.200 |  | 0.461 |  | 3.425 |  | 6.190e+33 |  | 1.666 |  |
| Time + group + Time ✻  group |  | 0.200 |  | 0.283 |  | 1.578 |  | 3.795e+33 |  | 4.810 |  |
| Time + group |  | 0.200 |  | 0.256 |  | 1.375 |  | 3.433e+33 |  | 1.747 |  |
| group |  | 0.200 |  | 2.169e-35 |  | 8.676e-35 |  | 0.291 |  | 1.049 |  |
|  | | | | | | | | | | | |
|  | | | | | | | | | | | |

Post-hoc Bayesian t-tests similarly showed extreme evidence for the differences between the different time points, see Table S2.

| Table S2. Bayesian t-test post-hoc comparisons for number of correct sequences performed (by group) | | | | | | | | | | | |
| --- | --- | --- | --- | --- | --- | --- | --- | --- | --- | --- | --- |
|  | |  | | **Prior Odds** | | **Posterior Odds** | | **BF_10, U_** | | **error %** | |
| Pretest |  | Posttest |  | 0.587 |  | 2.888e+16 |  | 4.917e+16 |  | 9.948e-22 |  |
|  |  | 24h |  | 0.587 |  | 5.831e+21 |  | 9.927e+21 |  | 5.749e-26 |  |
| Posttest |  | 24h |  | 0.587 |  | 2844.212 |  | 4842.027 |  | 5.618e-6 |  |
|  | | | | | | | | | | | |
|  | | | | | | | | | | | |

**

*Figure S1 – number of correct sequences performed for each of the four trials during the relevant test. Each color represents a different group. The faint dots are the values for all subjects, the darker lines and error bars are the mean ± standard error. *** represents p<=0.001*

*SM-2. Standard deviation of reaction times in training across groups*

To complement the analysis of coefficient of variation (standard deviation divided by mean), we also present the results of analyzing the standard deviation, see figure S2.

*Figure S2 – standard deviation of time between touching the thumb and the other finger, for the three groups, averaged across movements in the different blocks. The faint circles are data for all subjects, dark dots are the mean, error bars are the standard error for each group. The black bars indicate significant between-group differences, the red bars indicate significant differences between sessions. * represents p<=0.05, ** represents p<=0.01, *** represents p<=0.001*

We found a main effect of session (F(3,162)=27.423, p<0.001), post-hoc t-tests showed that each session was significantly different from all other sessions (all p<=0.012). Specifically the mean of the standard deviation reduced as a function of time: Pretest: 0.116 *±* 0.043 s, Training: 0.098 *±* 0.05 s, Posttest: 0.086 *±* 0.028 s 24h: 0.071 *±* 0.24s. A main effect of group was also observed (F(2,54)=3.583, p=0.035), post-hoc t-tests showed that the standard deviation for the Unsupervised control group (0.083 *±* 0.25) was lower than that of the variable group (0.105 *±* 0.024, p=0.033), the other differences were not significant. Additionally, there was an interaction of group and session: in the training session, the Variable group (0.143 *±* 0.019 s) had a significantly higher standard deviation that both the Fixed control (0.080 *±* 0.008, p<0.001) and Unsupervised control groups (0.073 *±* 0.017, p<0.001), but the Fixed control and Unsupervised control groups were not significantly different from each other.
